# Sleep duration predicts subsequent long-term mortality in patients with type 2 diabetes: a large single-center cohort study

**DOI:** 10.1186/s12933-022-01500-0

**Published:** 2022-04-27

**Authors:** Chia-Ing Li, Cheng-Chieh Lin, Chiu-Shong Liu, Chih-Hsueh Lin, Shing-Yu Yang, Tsai-Chung Li

**Affiliations:** 1 School of Medicine, College of Medicine, China Medical University, Taichung, Taiwan; 2 Department of Medical Research, China Medical University Hospital, Taichung, Taiwan; 3 Department of Family Medicine, China Medical University Hospital, Taichung, Taiwan; 4 Department of Public Health, College of Public Health, China Medical University, No. 100, Sec. 1, Jingmao Rd., Beitun Dist., Taichung, 406040 Taiwan; 5 Department of Healthcare Administration, College of Medical and Health Science, Asia University, Taichung, Taiwan

**Keywords:** Sleep duration, Type 2 diabetes, Mortality, Cardiovascular disease, Metabolic

## Abstract

**Background:**

Sleep duration is associated with mortality. However, prior studies exploring whether sleep duration predicts subsequent long-term mortality in patients with diabetes are limited. This study aims to examine whether metabolic factors affect the associations between baseline sleep duration and subsequent risks of all-cause, expanded, and non-expanded cardiovascular disease (CVD) mortalities among patients with type 2 diabetes (T2D).

**Methods:**

A total of 12,526 T2D patients aged 30 years and older, with a follow-up period ≥ 3 years, were identified from the Diabetes Case Management Program of a medical center in Taiwan. Sleep duration was measured using computerized questionnaires by case managers, and the time frame for this question was 1 month prior to the interview date. Sleep duration in relation to subsequent mortality from all causes, expanded CVD, and non-expanded CVD was examined using Cox proportional hazard models.

**Results:**

Within 10 years of follow-up, 2918 deaths (1328 CVD deaths and 1590 non-CVD deaths) were recorded. A J-shaped association was observed for all-cause, expanded CVD, and non-expanded CVD mortalities, and the lowest risks were observed for patients with 5–7 h of sleep. The significant joint effects included sleep duration of more or less than 7 h with age ≥ 65 years [adjusted HRs: 4.00 (3.49–4.60)], diabetes duration ≥ 5 years [1.60 (1.40–1.84)], age at diabetes diagnosis ≤ 45 years [1.69 (1.38–2.07)], insulin use [1.76 (1.54–2.03)], systolic blood pressure/diastolic blood pressure > 130/85 mmHg [1.24 (1.07–1.43)], triglyceride ≥ 150 mg/dL [1.38 (1.22–1.56)], HbA1c ≥ 7% [1.31 (1.13–1.52)], and body mass index < 27 kg/m^2^ [1.31 (1.17–1.45)] for all-cause mortality.

**Conclusion:**

A J-shaped association was observed between sleep duration and all-cause and expanded CVD mortality, and a sleep duration of 5–7 h had the lowest mortality risk. Sleep duration also showed significant synergistic interactions with diabetes duration but shared an antagonistic interaction with age and obesity.

**Supplementary Information:**

The online version contains supplementary material available at 10.1186/s12933-022-01500-0.

## Background

Sleep occupies approximately one-third of the day of most people. According to the National Sleep Foundation (NSF) in the USA, sleep is essential for a person’s health and wellbeing [1]. NSF recommends 7–9 and 7–8 h of sleep for adults aged 18–64 years and older adults ≥ 65 years, respectively [1]. Sleep disorders have been linked with a number of health problems, including obesity [2, 3], diabetes [4], cardiometabolic disease [2, 5–7], and mortality [8–10]. Itani and Jike et al. used the same methodology to explore the effects of sleep duration on different health outcomes in adults aged ≥ 20 years [11, 12]. They found that deviations from optimal sleep duration pose a substantial threat to health. A long sleep duration is significantly associated with pooled adjusted risk ratios (RRs) of 1.08 for obesity, 1.24 for coronary heart disease, 1.25 for cardiovascular disease (CVD), 1.26 for diabetes mellitus, and 1.39 for mortality [12]. The corresponding pooled adjusted RRs for short sleepers compared with normal sleepers are 1.38, 1.26, 1.16, 1.37, and 1.12 [11]. Previous studies provided different definitions of long and short sleep, but most studies defined long sleep as having a duration greater than 8 or 9 h per day and short sleep as having a duration less than 5 or 6 h per day [11, 12].

Good-quality sleep with adequate hours plays an effective role in cognitive performance [13] and preventing chronic health conditions, CVD, and even immature death [14]. Sleep difficulties have been reported as a long-COVID 19 sequelae [15]. Sleep is a critical component of healthy lifestyles, especially among patients with diabetes [16]. It is important for persons with type 2 diabetes (T2D) because they are susceptible to developing micro- and macro-vascular diseases [17], and sleep disorders affect blood sugar and insulin levels [18, 19]. A recent study reported sleep disturbance is significantly associated with an increased risk of CVD and all-cause mortality in patients with new-onset T2D [20], and a long sleep duration (≥ 9 h/day) has been shown to be associated with an increased all-cause mortality in persons with T2D [21]. A higher level of HbA1c can be found in patients with diabetes with short and long sleep durations than in those with medium sleep duration [22]. A review article linked the epidemiologic and lab evidence of physiological mechanisms by which insufficient sleep and sleep disorders increase the risk of diabetes [23]. A cross-sectional study reported that people with less than 5 h of sleep had two times higher prevalence of T2D than those with 7–9 h of sleep [24]. An epidemiologic cohort study found that sleep problems are associated with an increased likelihood of incident T2D [25]. Two population-based studies reported that short sleep duration is more common among persons with diabetes (37%) [26] than among the general population (24%) [7]. Furthermore, T2D may affect sleep. Higher prevalence rates of insomnia (50% vs. 31%) and sleeping pill use (26% vs. 6%) and lower sleep quality level were found in persons with diabetes than in those without diabetes [27]. However, the effects of sleep duration on other adverse health outcomes among patients with T2D are unclear. Related research on persons with T2D focused on short-term outcomes, such as glucose level. A recent study has found that variability in sleep duration within a week is the most important modifiable factor associated with sleep for HbA1c level in diabetes care [22], followed by the mean total sleep duration within a week. However, limited evidence is available regarding the effects of sleep duration on long-term outcomes, such as mortality, in patients with T2D.

Recent clinical evidence has suggested that sleep duration affects mortality [9, 28–33] in various populations. Most prior studies were conducted in the general population [9, 31, 32] and middle-aged and elderly [28, 29]. Three recent studies have examined the associations between sleep duration and mortality in patients with diabetes [21, 30, 33]. Although they all found a J-shaped association between sleep duration and all-cause mortality in patients with T2D, two studies reported increased risk of death in short (≤ 5 h) and long (≥ 9 h) sleep durations [30, 33], and one study did not observe the significant risk of death in short sleep duration [21]. In addition to the inconsistent findings on the independent effect of sleep, there existed conflict results on the effect modification of sleep duration with diabetes-related factors. Both Wang et al. [33] and Gu et al. [21] explored the interaction of sleep duration with age at diagnosis, diabetes duration, and type of anti-diabetes treatment. Wang’s study conducted in the US, reported significant interaction of the aforementioned three factors and sleep duration on mortality [33]. On the contrary, Gu’s study conducted in Korea, did not observe such interaction effects [21]. Furthermore, the interactions of sleep duration with some metabolic factors of hyperlipidemia and obesity have not been assessed. Therefore, the present study aimed to examine the associations of baseline sleep duration with subsequent risks of all-cause, expanded CVD, and non-expanded CVD mortality among Chinese persons with T2D interacted with age, diabetes duration, early onset of diabetes mellitus, insulin use, hypertension, hyperlipidemia, HbA1c, and obesity. Three additive interaction measures of relative excess risk due to interaction (RERI), the proportion attributable to interaction (AP), and the synergy index (S index) were reported to indicate the direction of interaction.

## Methods

### Study participants and data sources

A retrospective cohort study was carried out among persons who registered in the Diabetes Care Management Program (DCMP) of China Medical University Hospital (CMUH). DCMP is a case management program set up by the National Health Insurance Administration in 2001. Participants included those who were diagnosed with T2D in accordance with the criteria of American Diabetes Association (International Classification Disease, Ninth Revision, Clinical Modification, abbreviated as ICD-9-CM; code 250). The eligibility criterion of the retrospective cohort study was all enrollees in the DCMP registry between November 2001 and April 2016. Under the inclusion criteria, this cohort was open or dynamic, i.e., each eligible person was allowed to enter this study at different time points. Persons who can provide at least a 3-year follow-up period were also included to ensure that the follow-up period was sufficiently long. The exclusion criteria included persons with gestational diabetes (ICD-9-CM code 648.83) or type 1 diabetes (ICD-9-CM code 250. × 1/ × 3, *n* = 448), persons under the age of 30 years (*n* = 504), and persons who followed up < 3 years to rule out the possibility of reverse causality (*n* = 1007). The exclusion criteria enhanced the homogeneity of the study population to increase the internal validity of the study findings. A total of 16,414 enrolled patients with T2D met the above criteria for a retrospective cohort study. Finally, 12,526 patients were included in the analysis after those without information on sleep and baseline characteristics were excluded (Additional file 1: Fig. S1).

The computerized database of persons with T2D registered in the DCMP of CMUH provided information on lifestyle behaviors, vital signs, annual eye examinations, annual self-care education and assessment, and laboratory test results. The DCMP contained information on lifestyle behaviors, such as sleep duration, alcohol drinking, smoking, dietary habits, and regular exercise. Laboratory tests involved HbA1c, fasting plasma glucose (FPG), serum creatinine, triglyceride (TG), high density lipoprotein-cholesterol (HDL-C), low density lipoprotein-cholesterol (LDL-C), and total cholesterol (TC). The medication used included insulin, oral hypoglycemic agents, cholesterol lowering agents (e.g., statins [HMG-CoA reductase inhibitors]), and hypertension medications (e.g., calcium channel blockers). This study was approved by the Ethical Review Board of CMUH (CMUH109-REC1-197).

### Measurements

The enrollees underwent medical tests for vital signs, anthropometric measurements, blood, urine, and lifestyle behaviors. Medical history of previous or current disease status was also obtained by a case management nurse through a standardized computerized questionnaire upon entering the DCMP. Vital signs, anthropometric measurements, medical history, and lifestyle behaviors were recorded annually. The variables used in this study are shown below.

#### Sleep duration and sleep-related variables

Sleep duration was assessed with the question “What are typical hours of sleep in a 24-h period?” The time frame for this question was a month prior to the interview date. This study used the baseline sleep duration that was measured on the index date, i.e., the entry date for a subject to the DCMP by a care manager with a computerized questionnaire. Various cutoff points for sleep duration have been reported in several studies [9, 28–33], and the cutoff points for six classes were adopted as follows: ≤ 4, 5–6, 7, 8, 9–10, and > 10 h. The cutoff points were determined with a slight modification from two meta-analyses of prospective studies [9, 31]. The diagnostic criteria of obstructive sleep apnea (OSA) and sleep disorders were ascertained through electronic outpatient records by the ICD-9-CM diagnosis codes for diagnosis of OSA (ICD-9-CM codes 327.23, 780.51, 780.53, and 780.57) and sleep disorders (291.82, 292.85, 307.4, 307.41, 307.42, 307.45, 307.46, 307.47, 307.48, 307.49, 327.11, 327.12, 327.2, 327.21, 327.24, 327.26, 327.27, 327.29, 327.3, 327.31, 327.32, 327.33, 327.34, 327.35, 327.36, 327.37, 327.39, 327.42, 327.43, 327.52, 327.53, 327.59, 327.8, 333.94, 347.0, 347.00, 347.01, 347.10, 347.11, 780.5, 780.55, 780.56, 780.58, and 780.59). The hypnotic-sedative medications comprised benzodiazepine (BZD), non-BZD (or Z-drug), anti-depressant (trazodone, mirtazapine, and doxepin), and anti-psychotics (sulpiride, olanzapine, clothiapine, chlorpromazine, risperidone, and quetiapine).

#### Anthropometric measurements

Anthropometric measurements included weight, height, body mass index (BMI), systolic blood pressure (SBP)/diastolic blood pressure (DBP), and pulse pressure. The weight and height of an individual were measured using an auto-anthropometer (super-view, HW-666). The participants were asked to remove their shoes and wear light clothing during the measurement. BMI was calculated as weight (kg)/(height)^2^ (m^2^). Blood pressure was measured for SBP and DBP by using an electronic device (OMRON, HEM-770A, Japan).

#### Laboratory examination

A 12-h overnight fasting was required before blood collection, and the samples were sent for analysis within 4 h after blood collection. The serum levels of creatinine, LDL-C, HDL-C, TG, and TC were measured by a biochemical auto-analyzer (Beckman Coulter Synchron System, AU5800, Fullerton, CA, USA) at the Clinical Laboratory Department of a medical center. Whole-blood HbA1c level was measured through boronate-affinity high-performance liquid chromatography (Premier Hb9210™, Trinity Biotech Plc, IDA business Park, Ireland) assay (reference range, 4.6–6.5%) with inter- and intra-assay coefficient variations (CVs) of 1.50–1.62% and 0.85–1.62%, respectively. The linearity was from 3.8% HbA1c to 18.5% HbA1c to ensure accuracy for the whole population with diabetes. FPG was measured in blood obtained using NaF tubes, each of which contained 5 mg of sodium fluoride to inhibit glucose metabolism and 4 mg of potassium oxalate to chelate calcium and prevent coagulation. Inter- and intra-assay CVs were both at 4%. TC and TG were measured in serum. TG levels were determined by an enzymatic colorimetric method with inter- and intra-assay CVs of 6.8% and 5%, respectively. LDL-C and HDL-C levels were measured using a direct method with inter- and intra-assay CVs of 4.5% and 3% for LDL-C, respectively, and 4.5% for HDL-C. Estimated glomerular filtration rate (eGFR) was derived based on serum creatinine levels in accordance with the following Chronic Kidney Disease Epidemiology Collaboration equation [34]: eGFR (mL/min/1.73 m^2^) = 141 $\times$ min (Scr/κ,1)^α^ $\times$ max (Scr/κ,1)^−1.209^ $\times$ 0.993^age^ $\times$ 1.018 (if female) $\times$ 1.159 (if black); Scr: serum creatinine, κ: 0.7 for females and 0.9 for males, α: − 0.329 for females and − 0.411 for males. In this equation, min stands for the minimum of Scr/κ or 1, and max stands for the maximum of Scr/κ or 1.

#### Medication-related variable

The variables for pharmacologic agent use were derived from the DCMP dataset. The anti-diabetic drugs contained insulin and oral hypoglycemic agents, such as metformin, sulfonylurea, thiazolidinedione, meglitinide, and biguanide. Other medication-related variables consisted of hypertension, cardiovascular, and hyperlipidemia medications. All of these medications were each divided into two groups (yes and no).

The medications for hypertension included angiotensin-converting enzyme inhibitor, angiotensin II receptor blockers, calcium channel blockers, β-blockers, α-blockers, and diuretics. The medications for CVD included antiarrhythmic, anti-coagulants, antiplatelet, digoxin, and nitrates. The medications for hyperlipidemia consisted of fibrates (including bezafibrate, clofibrate, gemfibrozil, and fenofibrate), statins (including atorvastatin, fluvastatin, lovastatin, pitavastatin, pravastatin, rosuvastatin, and simvastatin), and ezetimibe.

#### Comorbidities and diabetic complications

Baseline metabolic morbidities consisted of hypertension and hyperlipidemia. Baseline diabetic acute complications included hyperglycemic hyperosmolar nonketotic coma (HHNK), severe hypoglycemia, and diabetic ketoacidosis (DKA). Chronic complications were stroke, coronary artery diseases, severe hypoglycemia, and peripheral neuropathy. All of these diabetic comorbidities were each divided into two groups (yes and no).

### Outcome measures

The primary outcomes were total deaths and deaths from expanded and non-expanded CVD diseases, which were identified from the annual record linkage with the National Death Datasets provided by Taiwan Ministry of Health and Welfare by using basic information (personal identification number and date of birth). All patients were followed up from the index date to August 2021 or withdrawal from the DCMP or death. The index date was the entry date into the DCMP. The underlying cause of death was classified in accordance with the rules of ICD-9-CM from 2006 to 2008 and the International Classification of Diseases, Tenth Revision, Clinical Modification (ICD-10-CM) from 2009 to 2021. Expanded CVD mortality was defined as death from CVD (ICD-9-CM codes 390–459, ICD-10-CM codes I00–I99), plus diabetes (ICD-9-CM code 250, ICD-10-CM codes E10–E14), and plus kidney diseases (ICD-9-CM 580–589; ICD-10-CM N00–N29), whereas non-expanded CVD mortality was defined as total deaths not from expanded CVD causes [35].

### Statistical analysis

Simple descriptive analyses of proportion were performed for categorical variables and mean with standard deviation for continuous variables. Then, mortality cumulative incidence was estimated using the Kaplan–Meier (K–M) approach, presented as plots. Log-rank tests were applied to examine the differences in K–M mortality curves between subgroups of baseline sleep characteristics. Multivariate Cox proportional hazard models were used to estimate the hazard ratios (HRs) and their 95% confidence intervals (CIs). The assumption of proportionality was tested by entering an interaction (product) term for variables of sleep characteristics and person–time in the Cox models. The *p* for linear trend was reported for ordinal variables of sleep characteristics. Restricted cubic splines were used in the Cox models to examine the presence of a dose response or non-linear association of sleep duration as continuous variable with mortality. In addition, sensitivity analyses were performed to evaluate whether the findings of this study were similar under two conditions. One condition is that when patients with stroke, severe hypoglycemia, HHNK syndrome, and DKA were excluded for assessing the impact of comorbidities and the other condition is to include patients aged under 30 years as the proportion of this subgroup is increasing.

The interaction of sleep duration with age, diabetes-related factors, metabolic factors, and obesity in association with mortality was examined using three measures of additive interaction: RERI, AP, and S index [36]. In the absence of additive interaction, RERI and AP are equal to 0, and the S index is equal to 1. In the presence of a negative interaction, PERI or AP < 0 and S index < 1. In the presence of a positive interaction, PERI or AP > 0 and S index > 1. The analyses were performed with SAS version 9.4 (SAS, Cary, NC). Two-tailed *p*-values were considered, and a *p*-value < 0.05 indicated statistical significance.

## Results

Of the 12,526 participants enrolled in the DCMP, 2918 died (including 1328 expanded CVD deaths and 1590 non-expanded CVD deaths) during the mean 10.82 years of follow-up (standard deviation of 3.90 years). Table 1 presents the associations between sleep duration categories (≤ 4, 5–6, 7, 8, 9–10, and > 10) and baseline sociodemographic factors, lifestyle behaviors, diabetes-related variables, and complications. The patients who slept for more than 10 h/day had high proportions in female gender, non-alcohol drinking, physical inactive, age at diabetes diagnosis > 45 years, combination of oral hypoglycemic drug and insulin use, hypertension, stroke, coronary artery disease, peripheral neuropathy, hypertension medication use, and cardiovascular medication use than patients who slept for 7 h/day. On the contrary, they had lower mean values in BMI and eGFR but higher mean values in age, diabetes duration, HbA1c, FPG level, TG, and TC than patients who slept for 7 h/day. In addition, those who slept for less than and equal to 4 h/day had higher proportions in sleep disorders and hypnotic-sedative medications than patients who slept 7 h/day.

**Table 1:** Comparisons of baseline sociodemographic factors, lifestyle behaviors, diabetes-related variables, and complications according to sleep duration in patients with type 2 diabetes (n = 12,526)

Variables	Sleep duration (h/day)						
	≤ 4 (n = 205)	5–6 (n = 2,009)	7 (n = 3,276)	8 (n = 3,673)	9–10 (n = 2,910)	> 10 (n = 453)	*p* value
*Sociodemographic factors*							
Sex							< 0.001
Men	105 (51.22)	1025 (51.02)	1775 (54.18)	2028 (55.21)	1448 (49.76)	220 (48.57)	
Women	100 (48.78)	984 (48.98)	1501 (45.82)	1645 (44.79)	1462 (50.24)	233 (51.43)	
Age (years)†	58.97 ± 11.36	58.19 ± 11.13	57.79 ± 11.37	57.74 ± 11.64	60.80 ± 12.51	63.80 ± 13.51	< 0.001
*Lifestyle behaviors*							
Smoking	48 (23.41)	347 (17.27)	581 (17.74)	686 (18.68)	478 (16.43)	81 (17.88)	0.06
Alcohol drinking	23 (11.22)	172 (8.56)	294 (8.97)	319 (8.68)	197 (6.77)	29 (6.40)	0.006
Exercising	82 (40.00)	1073 (53.41)	1797 (54.85)	1922 (52.33)	1297 (44.57)	153 (33.77)	< 0.001
BMI (kg/m^2^)^†^	26.31 ± 4.32	26.28 ± 4.05	25.99 ± 4.15	25.88 ± 4.10	25.68 ± 4.16	25.38 ± 3.91	< 0.001
*Diabetes-related variables*							
Duration of diabetes (years)†	5.31 ± 6.25	5.53 ± 6.62	5.46 ± 6.49	5.42 ± 6.51	6.50 ± 7.28	7.89 ± 8.45	< 0.001
Age at diabetes diagnosis > 45 years	150 (73.17)	1463 (72.82)	2351 (71.76)	2606 (70.95)	2177 (74.81)	356 (78.59)	< 0.001
Types of diabetes treatment							< 0.001
Diet or exercise	14 (6.83)	138 (6.87)	241 (7.36)	248 (6.75)	158 (5.43)	17 (3.75)	
Oral hypoglycemic drug	163 (79.51)	1658 (82.53)	2695 (82.26)	3061 (83.34)	2377 (81.68)	373 (82.34)	
Insulin injection	3 (1.46)	28 (1.39)	40 (1.22)	51 (1.39)	50 (1.72)	5 (1.10)	
Both	25 (12.2)	185 (9.21)	300 (9.16)	313 (8.52)	325 (11.17)	58 (12.8)	
*Complications*							
Hypertension	101 (49.27)	947 (47.14)	1430 (43.65)	1633 (44.46)	1388 (47.70)	235 (51.88)	< 0.001
Hyperlipidemia	58 (28.29)	494 (24.59)	797 (24.33)	873 (23.77)	684 (23.51)	93 (20.53)	0.29
Stroke	16 (7.8)	69 (3.43)	94 (2.87)	122 (3.32)	188 (6.46)	49 (10.82)	< 0.001
Coronary artery disease	49 (23.9)	351 (17.47)	566 (17.28)	645 (17.56)	646 (22.20)	119 (26.27)	< 0.001
Severe hypoglycemia	2 (0.98)	11 (0.55)	22 (0.67)	31 (0.84)	26 (0.89)	6 (1.32)	0.49
Peripheral neuropathy	25 (12.2)	195 (9.71)	263 (8.03)	319 (8.68)	333 (11.44)	55 (12.14)	< 0.001
DKA	0 (0.00)	12 (0.60)	13 (0.40)	14 (0.38)	11 (0.38)	1 (0.22)	0.68
HHNK	4 (1.95)	15 (0.75)	30 (0.92)	30 (0.82)	40 (1.37)	6 (1.32)	0.10
Sleep disorders	47 (22.93)	387 (19.26)	530 (16.18)	590 (16.06)	575 (19.76)	79 (17.44)	< 0.001
OSA	3 (1.46)	25 (1.24)	41 (1.25)	35 (0.95)	33 (1.13)	8 (1.77)	0.64
*Medication use*							
Hypertension medications	87 (42.44)	840 (41.81)	1253 (38.25)	1447 (39.40)	1231 (42.30)	216 (47.68)	< 0.001
Hyperlipidemia medications	30 (14.63)	249 (12.39)	427 (13.03)	467 (12.71)	372 (12.78)	56 (12.36)	0.95
Cardiovascular medications	41 (20.00)	325 (16.18)	524 (16)	599 (16.31)	591 (20.31)	102 (22.52)	< 0.001
Hypnotic-sedative medications	71 (34.63)	510 (25.39)	739 (22.56)	883 (24.04)	831 (28.56)	154 (34.00)	< 0.001
*Biomarkers*†							
HbA1c (%)	8.23 ± 1.99	7.99 ± 1.76	7.89 ± 1.76	7.88 ± 1.78	7.94 ± 1.86	8.03 ± 1.94	0.02
FPG (mg/dL)	159.76 ± 69.04	154.16 ± 56.35	152.25 ± 52.85	151.60 ± 51.09	155.45 ± 60.98	160.75 ± 69.63	0.001
LDL-C (mg/dL)	116.13 ± 39.97	112.57 ± 35.39	112.17 ± 33.49	111.54 ± 34.58	112.53 ± 36.37	114.81 ± 35.72	0.23
HDL-C (mg/dL)	43.23 ± 10.32	43.08 ± 11.51	43.26 ± 11.38	43.10 ± 11.42	43.12 ± 12.22	42.33 ± 12.04	0.75
TC (mg/dL)	192.87 ± 48.75	187.59 ± 40.93	187.53 ± 40.81	186.60 ± 41.13	189.97 ± 45.49	193.24 ± 42	0.001
TG (mg/dL)	169.49 ± 158.61	159.3 ± 132.7	156.36 ± 145.27	156.62 ± 136.58	171.45 ± 169.22	183.34 ± 169.8	< 0.001
eGFR (ml/min/1.73m^2^)	85.30 ± 23.40	86.80 ± 22.52	87.28 ± 22.56	86.54 ± 23.15	79.72 ± 26.58	75.44 ± 26.94	< 0.001

ANOVA was used for continuous variables to calculate *p*-values

Chi-square test was used for categorical variables to calculate *p*-values

^†^Mean ± SD; CKD: chronic kidney disease; BMI: body mass index: DKA: diabetic ketoacidosis; HHNK: hyperglycemic hyperosmolar nonketotic coma; OSA: obstructive sleep apnea; FPG: fasting plasma glucose; LDL-C: low-density lipoprotein-cholesterol; HDL-C: high-density lipoprotein-cholesterol; TG: triglyceride; TC: total cholesterol; eGFR: estimated glomerular filtration rate

The prevalence rates of self-reported sleep durations of ≤ 4, 5–6, 7, 8, 9–10, and > 10 h*/*day were 1.64%, 16.04%, 26.15%, 29.32%, 23.23%, and 3.62%, respectively. The associations of sleep duration categories with all-cause, expanded CVD, and non-expanded CVD mortalities are reported in Table 2. Multivariate Cox regression analysis showed that patients with sleep durations of ≤ 4, 9–10, and > 10 h*/*day had significantly higher mortality risks than those with a sleep duration of 7 h*/*day (HR = 1.41, 95% CI 1.06–1.86; 1.37, 1.23–1.52, and 1.82, 1.54–2.14, respectively). For expanded CVD mortality, patients who slept for ≤ 4, 9–10, and > 10 h*/*day had higher risks than those who slept for 7 h/day (HR 1.54, 95% CI 1.04–2.28; 1.34, 1.15–1.57; and 1.88, 1.49–2.37, respectively). For non-expanded CVD mortality, the excess risks were observed among patients with sleep durations of 8, 9–10, and > 10 h*/*day (HR 1.16, 95% CI 1.01–1.34; 1.39, 1.20–1.60; and 1.77, 1.41–2.23, respectively).

**Table 2:** Hazard ratios of mortality for sleep duration in patients with type 2 diabetes (n = 12,526)

*Sleep duration (h/day)*	n	case	Person-years	Incidence rate^†^	Multivariate model 1		Multivariate model 2		Multivariate model 3	
					HR (95% CI)	*p* value	HR (95% CI)	*p* value	HR (95% CI)	*p* value
*All-cause mortality*										
≤ 4	205	55	2232.16	24.64	1.54 (1.17, 2.03)	0.002	1.42 (1.08, 1.88)	0.01	1.41 (1.06, 1.86)	0.02
5–6	2009	421	22,717.92	18.53	1.07 (0.94, 1.21)	0.30	1.07 (0.95, 1.21)	0.29	1.06 (0.94, 1.20)	0.35
7	3276	608	36,629.43	16.60	1.00		1.00		1.00	
8	3673	750	39,990.38	18.75	1.15 (1.03, 1.28)	0.01	1.14 (1.02, 1.26)	0.02	1.10 (0.99, 1.23)	0.07
9–10	2910	879	29,810.22	29.49	1.59 (1.43, 1.76)	< 0.001	1.48 (1.33, 1.64)	< 0.001	1.37 (1.23, 1.52)	< 0.001
> 10	453	205	4155.32	49.33	2.29 (1.95, 2.68)	< 0.001	2.03 (1.73, 2.38)	< 0.001	1.82 (1.54, 2.14)	< 0.001
*Expanded CVD mortality*										
≤ 4	205	29	2232.16	12.99	1.78 (1.21, 2.61)	0.003	1.65 (1.12, 2.42)	0.01	1.54 (1.04, 2.28)	0.03
5–6	2009	185	22,717.92	8.14	1.04 (0.86, 1.25)	0.71	1.04 (0.86, 1.25)	0.71	1.02 (0.85, 1.23)	0.84
7	3276	273	36,629.43	7.45	1.00		1.00		1.00	
8	3673	319	39,990.38	7.97	1.10 (0.93, 1.29)	0.27	1.09 (0.93, 1.28)	0.31	1.04 (0.89, 1.23)	0.61
9–10	2910	418	29,810.22	14.02	1.69 (1.45, 1.97)	< 0.001	1.56 (1.33, 1.82)	< 0.001	1.34 (1.15, 1.57)	< 0.001
> 10	453	104	4155.32	25.03	2.61 (2.07, 3.28)	< 0.001	2.29 (1.82, 2.88)	< 0.001	1.88 (1.49, 2.37)	< 0.001
*Non-expanded CVD mortality*										
≤ 4	205	26	2232.16	11.65	1.34 (0.90, 2.00)	0.15	1.24 (0.83, 1.85)	0.30	1.29 (0.86, 1.92)	0.22
5–6	2009	236	22,717.92	10.39	1.10 (0.93, 1.29)	0.29	1.10 (0.93, 1.30)	0.27	1.10 (0.93, 1.30)	0.27
7	3276	335	36,629.43	9.15	1.00		1.00		1.00	
8	3673	431	39,990.38	10.78	1.19 (1.03, 1.38)	0.02	1.18 (1.02, 1.36)	0.03	1.16 (1.01, 1.34)	0.04
9–10	2910	461	29,810.22	15.46	1.51 (1.31, 1.74)	< 0.001	1.42 (1.23, 1.63)	< 0.001	1.39 (1.20, 1.60)	< 0.001
> 10	453	101	4155.32	24.31	2.03 (1.62, 2.54)	< 0.001	1.82 (1.45, 2.28)	< 0.001	1.77 (1.41, 2.23)	< 0.001

^†^Number of incident cases/person-years*1000

Multivariate model 1 adjusted for age and sex

Multivariate model 2 additionally adjusted for smoking, alcohol drinking, exercising, body mass index, duration of diabetes, age at diabetes diagnosis, and types of diabetes treatment in the first multivariate model

Multivariate model 3 additionally adjusted for complications, medication use and biomarkers in addition to the variables in the second multivariate model

In Table 3, patients with stroke, severe hypoglycemia, diabetic ketoacidosis, and hyperglycemic hyperosmolar nonketotic coma were excluded in the sensitivity analysis (n = 11,748). In the sensitivity analysis, similar results were observed for the HRs of all-cause mortality (1.41, 1.04–1.92 for ≤ 4 h/day; 1.12, 1.00–1.25 for 8 h/day; 1.41, 1.26–1.57 for 9–10 h/day; and 1.88, 1.58–2.24 for ≥ 10 h/day), expanded CVD mortality (1.90, 1.26–2.86 for ≤ 4 h/day; 1.40, 1.18–1.66 for 9–10 h/day; and 1.99, 1.54–2.58 for ≥ 10 h/day, respectively), and non-expanded CVD mortality (1.17, 1.01–1.35 for 8 h/day; 1.40, 1.21–1.63 for 9–10 h/day; and 1.79, 1.41–2.28 for ≥ 10 h/day, respectively). By including those whose aged under 30 years, patients with sleep duration ≤ 4, 9–10, and > 10 h/day exhibited a higher risk of all-cause mortality and expanded CVD mortality (adjusted HR: 1.41, 1.06–1.86; 1.37, 1.24–1.52; and 1.83, 1.56–2.15 for all-cause mortality, and 1.53, 1.04–2.27; 1.34, 1.15–1.57; and 1.90, 1.50–2.39 for expanded CVD mortality, respectively) than those with sleep duration of 7 h/day after multivariate adjustment (Additional file 2: Table S1).

**Table 3:** Sensitivity analysis for bias due to the existence of comorbidities by excluding individuals with stroke, severe hypoglycemia, diabetic ketoacidosis, and hyperglycemic hyperosmolar nonketotic coma

*Sleep duration (h/day)*	Model I		Model II		Model III		Model IV		Model V	
	HR (95% CI)	*p* value	HR (95% CI)	*p* value	HR (95% CI)	*p* value	HR (95% CI)	*p* value	HR (95% CI)	*p* value
*All-cause mortality*										
≤ 4	1.47 (1.09, 1.99)	0.01	1.50 (1.13, 1.98)	0.005	1.40 (1.06, 1.86)	0.02	1.40 (1.05, 1.86)	0.02	1.41 (1.04, 1.92)	0.03
5–6	1.09 (0.96, 1.24)	0.20	1.06 (0.94, 1.21)	0.33	1.05 (0.93, 1.19)	0.44	1.07 (0.94, 1.21)	0.32	1.08 (0.95, 1.23)	0.25
7	1.00		1.00		1.00		1.00		1.00	
8	1.13 (1.01, 1.26)	0.03	1.09 (0.97, 1.21)	0.14	1.11 (1.00, 1.23)	0.06	1.12 (1.00, 1.25)	0.04	1.12 (1.00, 1.25)	0.05
9–10	1.42 (1.27, 1.58)	< 0.001	1.34 (1.20, 1.49)	< 0.001	1.37 (1.23, 1.52)	< 0.001	1.39 (1.25, 1.54)	< 0.001	1.41 (1.26, 1.57)	< 0.001
> 10	1.91 (1.61, 2.27)	< 0.001	1.80 (1.53, 2.12)	< 0.001	1.82 (1.54, 2.14)	< 0.001	1.82 (1.55, 2.15)	< 0.001	1.88 (1.58, 2.24)	< 0.001
*Expanded CVD mortality*										
≤ 4	2.00 (1.34, 2.98)	< 0.001	1.76 (1.19, 2.60)	0.005	1.54 (1.04, 2.28)	0.03	1.57 (1.05, 2.34)	0.03	1.90 (1.26, 2.86)	0.002
5–6	1.08 (0.88, 1.31)	0.47	1.02 (0.85, 1.23)	0.82	1.01 (0.84, 1.22)	0.90	1.03 (0.85, 1.24)	0.80	1.05 (0.86, 1.28)	0.66
7	1.00		1.00		1.00		1.00		1.00	
8	1.08 (0.91, 1.28)	0.38	1.01 (0.86, 1.19)	0.91	1.05 (0.89, 1.24)	0.54	1.07 (0.91, 1.26)	0.43	1.06 (0.89, 1.26)	0.51
9–10	1.44 (1.22, 1.70)	< 0.001	1.29 (1.11, 1.52)	< 0.001	1.35 (1.16, 1.58)	< 0.001	1.37 (1.17, 1.61)	< 0.001	1.40 (1.18, 1.66)	< 0.001
> 10	2.05 (1.59, 2.64)	< 0.001	1.85 (1.46, 2.34)	< 0.001	1.88 (1.49, 2.38)	< 0.001	1.91 (1.51, 2.42)	< 0.001	1.99 (1.54, 2.58)	< 0.001
*Non-expanded CVD mortality*										
≤ 4	1.10 (0.70, 1.73)	0.69	1.31 (0.88, 1.96)	0.18	1.26 (0.85, 1.89)	0.25	1.22 (0.81, 1.86)	0.34	1.05 (0.66, 1.67)	0.83
5–6	1.10 (0.92, 1.30)	0.29	1.10 (0.93, 1.30)	0.28	1.08 (0.91, 1.28)	0.36	1.10 (0.93, 1.30)	0.27	1.11 (0.93, 1.31)	0.26
7	1.00		1.00		1.00		1.00		1.00	
8	1.17 (1.01, 1.35)	0.04	1.15 (0.99, 1.33)	0.06	1.16 (1.00, 1.34)	**0.05**	1.16 (1.01, 1.34)	0.04	1.17 (1.01, 1.35)	0.04
9–10	1.39 (1.20, 1.61)	< 0.001	1.37 (1.19, 1.58)	< 0.001	1.38 (1.19, 1.59)	< 0.001	1.39 (1.20, 1.61)	< 0.001	1.40 (1.21, 1.63)	< 0.001
> 10	1.81 (1.43, 2.29)	< 0.001	1.74 (1.38, 2.19)	< 0.001	1.75 (1.39, 2.2)	< 0.001	1.74 (1.38, 2.19)	< 0.001	1.79 (1.41, 2.28)	< 0.001

Multivariate model adjusted for age, sex, smoking, alcohol drinking, exercising, body mass index, duration of diabetes, age at diabetes diagnosis, types of diabetes treatment, complications, medication use, and biomarkers

Model I excluded persons with stroke (n = 538)

Model II excluded individuals with severe hypoglycemia (n = 98)

Model III excluded individuals with diabetic ketoacidosis (n = 51)

Model IV excluded individuals with hyperglycemic hyperosmolar nonketotic coma (n = 125)

Model V excluded individuals with stroke, coronary artery disease, severe hypoglycemia, diabetic ketoacidosis, and hyperglycemic hyperosmolar nonketotic coma (n = 778)

**Fig. 1:**
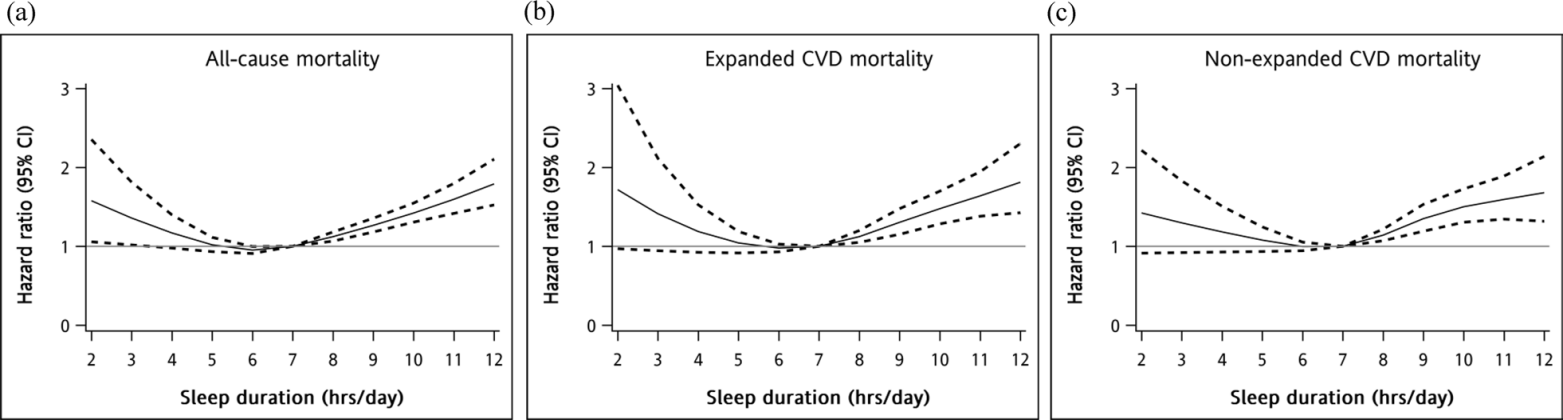
Multivariable cubic spline plots for **a** all-cause mortality, **b** expanded CVD mortality, and **c** non-expanded CVD mortality by sleep duration

**Fig. 2:**
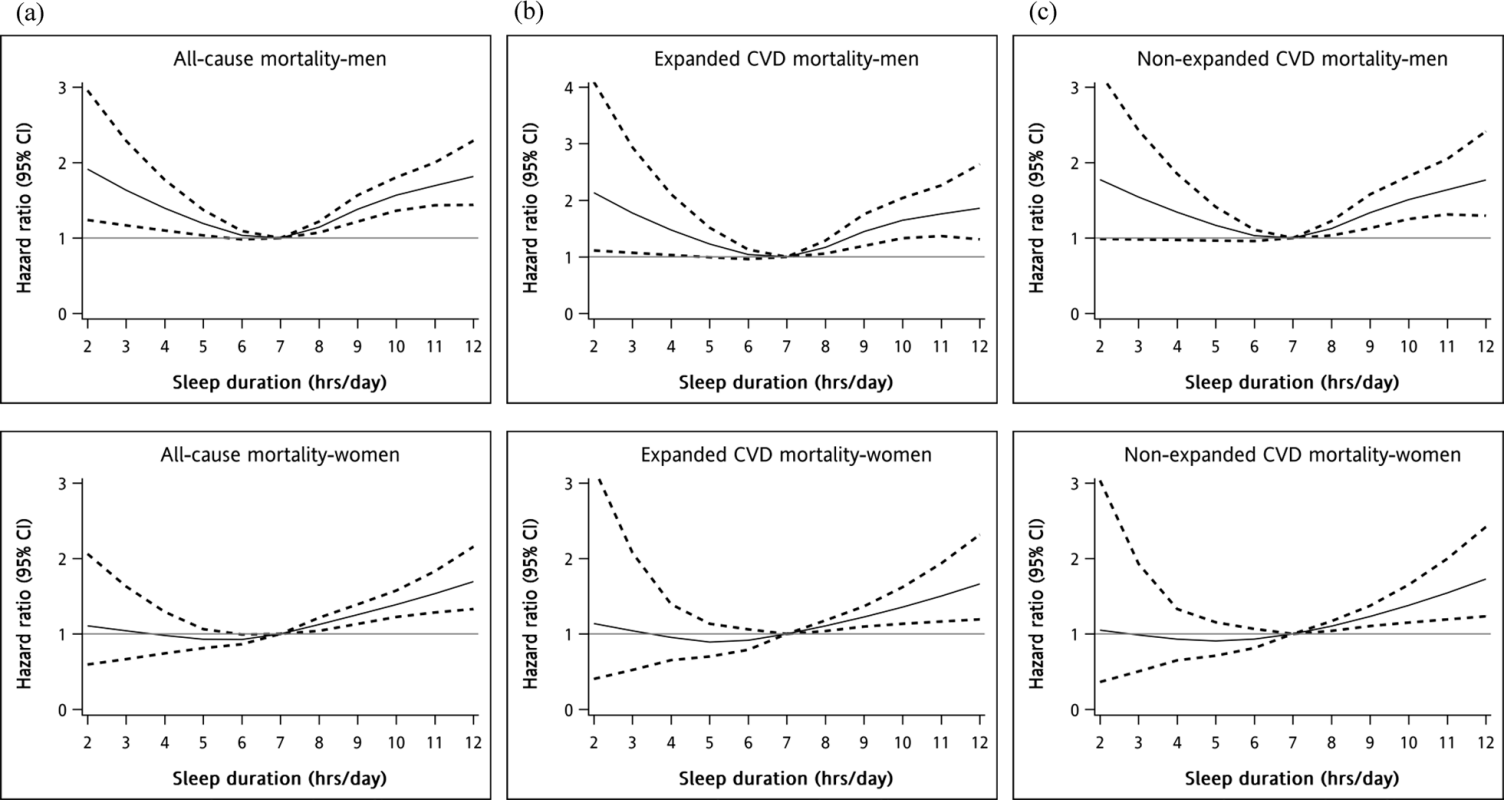
Multivariable cubic spline plots for **a** all-cause mortality, **b** expanded CVD mortality, and **c** non-expanded CVD mortality by sleep duration stratified by sex

The restricted multivariable cubic spline plots for all-cause, expanded CVD, and non-expanded CVD mortalities are presented in Fig. 1, demonstrating significant non-linear associations with the abovementioned mortalities. Significant interactions were found between sex and sleep duration (p for interaction = 0.03). Subgroup analysis showed different curves among women and men (Fig. 2). After adjustment for covariates, a negative association was found between sleep duration < 7 h and mortality in men but not in women.

The joint effects of age and diabetes-related and metabolic factors with sleep duration on all-cause, expanded CVD, and non-expanded CVD mortalities in the total sample were explored (Table 4). The adjusted HRs of sleep duration of more or less than 7 h with age ≥ 65 years, diabetes duration ≥ 5 years, age at diabetes diagnosis ≤ 45 years, insulin use, SBP/DBP > 130/85 mmHg, TG ≥ 150 mg/dL, HbA1c ≥ 7%, and BMI < 27 kg/m^2^ were all significant for all-cause, expanded CVD, and non-expanded CVD mortalities. Significant interactions were observed between sleep duration and obesity on all-cause mortality (p for interaction = 0.03), sleep duration and age on expanded CVD mortality (p for interaction = 0.04), and sleep duration and diabetes duration and obesity on non-expanded CVD mortality (p for interaction = 0.05 and 0.008, respectively).

**Table 4:** Adjusted HR of mortality for the effects of sleep duration with age, duration of diabetes, age at diabetes diagnosis, insulin use, hypertension, hyperlipidemia, HbA1c, and obesity

Variables	HR (95% CI)		
	All-cause mortality	Expanded CVD mortality	Non-expanded CVD mortality
Sleep duration = 7 & age < 65	1.00	1.00	1.00
Sleep duration = 7 & age ≥ 65	3.48 (2.95, 4.12)***	3.61 (2.81, 4.64)***	3.41 (2.72, 4.26)***
Sleep duration ≠ 7 & age < 65	1.32 (1.16, 1.51)***	1.40 (1.14, 1.71)**	1.27 (1.06, 1.51)**
Sleep duration ≠ 7 & age ≥ 65	4.00 (3.49, 4.60)***	3.82 (3.09, 4.72)***	4.20 (3.50, 5.04)***
*p* for interaction	0.12	0.04	0.82
RERI	0.20 (-0.26, 0.65)	-0.19 (-0.88, 0.51)	0.52 (-0.09, 1.14)
AP	0.05 (-0.08, 0.18)	-0.05 (-0.26, 0.16)	0.12 (-0.03, 0.28)
S index	1.07 (0.91, 1.26)	0.94 (0.74, 1.18)	1.20 (0.95, 1.50)
Sleep duration = 7 & duration of DM < 5	1.00	1.00	1.00
Sleep duration = 7 & duration of DM ≥ 5	1.24 (1.05, 1.46)**	1.55 (1.20, 1.99)***	1.05 (0.84, 1.30)
Sleep duration ≠ 7 & duration of DM < 5	1.15 (1.00, 1.32)*	1.24 (0.99, 1.54)	1.10 (0.93, 1.31)
Sleep duration ≠ 7 & duration of DM ≥ 5	1.60 (1.40, 1.84)***	1.82 (1.47, 2.27)***	1.47 (1.24, 1.75)***
*p* for interaction	0.25	0.72	0.05
RERI	0.21 (0.01, 0.41)	0.04 (− 0.32, 0.39)	0.32 (0.08, 0.56)
AP	0.13 (0.04, 0.23)	0.02 (− 0.15, 0.19)	0.22 (0.11, 0.33)
S index	1.55 (0.86, 2.78)	1.05 (0.66, 1.66)	3.17 (0.39, 25.92)
Sleep duration = 7 & DM diagnosis > 45	1.00	1.00	1.00
Sleep duration = 7 & DM diagnosis ≤ 45	1.39 (1.13, 1.72)**	1.00 (0.74, 1.34)	1.90 (1.39, 2.60)***
Sleep duration ≠ 7 & DM diagnosis > 45	1.29 (1.04, 1.59)*	1.14 (0.86, 1.51)	1.44 (1.05, 1.97)*
Sleep duration ≠ 7 & DM diagnosis ≤ 45	1.69 (1.38, 2.07)***	1.22 (0.93, 1.60)	2.32 (1.72, 3.12)***
*p* for interaction	0.63	0.69	0.34
RERI	0.01 (− 0.25, 0.28)	0.08 (− 0.24, 0.4)	− 0.02 (− 0.46, 0.42)
AP	0.01 (− 0.12, 0.14)	0.06 (− 0.09, 0.22)	− 0.01 (− 0.21, 0.19)
S index	1.02 (0.68, 1.51)	1.55 (0.09, 26.96)	0.98 (0.71, 1.36)
Sleep duration = 7 & non-insulin use	1.00	1.00	1.00
Sleep duration = 7 & insulin use	1.40 (1.12, 1.76)**	1.37 (0.99, 1.89)	1.43 (1.03, 1.98)*
Sleep duration ≠ 7 & non-insulin use	1.23 (1.11, 1.35)***	1.22 (1.05, 1.41)**	1.24 (1.09, 1.41)**
Sleep duration ≠ 7 & insulin use	1.76 (1.54, 2.03)***	1.57 (1.28, 1.93)***	1.93 (1.60, 2.33)***
*p* for interaction	0.79	0.76	0.55
RERI	0.13 (− 0.22, 0.49)	− 0.01 (− 0.49, 0.47)	0.26 (− 0.26, 0.78)
AP	0.08 (− 0.1, 0.25)	− 0.01 (− 0.27, 0.26)	0.14 (− 0.09, 0.37)
S index	1.21 (0.7, 2.11)	0.98 (0.43, 2.25)	1.39 (0.66, 2.94)
Sleep duration = 7 & SBP/DBP ≤ 130/85	1.00	1.00	1.00
Sleep duration = 7 & SBP/DBP > 130/85	0.96 (0.81, 1.13)	1.00 (0.78, 1.28)	0.92 (0.74, 1.15)
Sleep duration ≠ 7 & SBP/DBP ≤ 130/85	1.12 (0.97, 1.30)	1.11 (0.89, 1.39)	1.13 (0.93, 1.37)
Sleep duration ≠ 7 & SBP/DBP > 130/85	1.24 (1.07, 1.43)**	1.25 (1.01, 1.56)*	1.22 (1.01, 1.47)*
p for interaction	0.11	0.38	0.19
RERI	0.16 (− 0.01, 0.34)	0.14 (− 0.13, 0.41)	0.17 (− 0.06, 0.4)
AP	0.13 (0.04, 0.22)	0.11 (− 0.02, 0.25)	0.14 (0.03, 0.26)
S index	3.17 (0.11, 94.90)	2.32 (0.08, 68.01)	4.61 (0.003, 7316.63)
Sleep duration = 7 & TG < 150 mg/dL	1.00	1.00	1.00
Sleep duration = 7 & TG ≥ 150 mg/dL	1.09 (0.92, 1.29)	1.35 (1.05, 1.72)*	0.91 (0.72, 1.15)
Sleep duration ≠ 7 & TG < 150 mg/dL	1.21 (1.08, 1.35)**	1.23 (1.03, 1.47)*	1.19 (1.03, 1.38)*
Sleep duration ≠ 7 & TG ≥ 150 mg/dL	1.38 (1.22, 1.56)***	1.56 (1.29, 1.88)***	1.25 (1.06, 1.47)**
*p* for interaction	0.63	0.66	0.29
RERI	0.08 (v0.13, 0.28)	− 0.02 (− 0.36, 0.32)	0.14 (− 0.11, 0.39)
AP	0.06 (− 0.06, 0.17)	− 0.01 (− 0.2, 0.17)	0.11 (− 0.03, 0.26)
S index	1.25 (0.62, 2.52)	0.96 (0.54, 1.72)	2.36 (0.15, 36.82)
Sleep duration = 7 & HbAlc < 7%	1.00	1.00	1.00
Sleep duration = 7 & HbAlc ≥ 7%	1.04 (0.88, 1.24)	1.28 (0.98, 1.66)	0.91 (0.72, 1.13)
Sleep duration ≠ 7 & HbAlc < 7%	1.18 (1.01, 1.38)*	1.21 (0.94, 1.55)	1.18 (0.96, 1.44)
Sleep duration ≠ 7 & HbAlc ≥ 7%	1.31 (1.13, 1.52)***	1.53 (1.21, 1.93)***	1.17 (0.96, 1.41)
*p* for interaction	0.54	0.96	0.49
RERI	0.08 (− 0.11, 0.28)	0.04 (− 0.28, 0.37)	0.08 (− 0.17, 0.33)
AP	0.06 (− 0.04, 0.16)	0.03 (− 0.13, 0.19)	0.07 (− 0.06, 0.20)
S index	1.37 (0.50, 3.80)	1.09 (0.53, 2.23)	1.96 (0.05, 82.24)
Sleep duration = 7 & BMI < 27 kg/m^2^	1.00	1.00	1.00
Sleep duration = 7 & BMI ≥ 27 kg/m^2^	1.02 (0.86, 1.22)	0.85 (0.65, 1.10)	1.19 (0.95, 1.50)
Sleep duration ≠ 7 & BMI < 27 kg/m^2^	1.31 (1.17, 1.45)***	1.21 (1.03, 1.42)*	1.39 (1.20, 1.61)***
Sleep duration ≠ 7 & BMI ≥ 27 kg/m^2^	1.08 (0.95, 1.22)	0.99 (0.83, 1.19)	1.16 (0.98, 1.38)
*p* for interaction	0.03	0.85	0.008
RERI	− 0.25 (− 0.46, − 0.03)	− 0.06 (− 0.34, 0.22)	− 0.42 (− 0.75, − 0.09)
AP	− 0.23 (− 0.39, − 0.07)	− 0.06 (− 0.26, 0.13)	− 0.36 (− 0.61, − 0.12)
S index	0.24 (0.06, 0.93)	− 0.13 (NE, NE)	0.27 (0.10, 0.73)

SBP: systolic blood pressure; DBP: diastolic blood pressure; TG: triglyceride; BMI: body mass index; AP: proportion attributable to interaction; RERI: relative excess risk due to interaction; S index: synergy index; *p < 0.05; **p < 0.01; ***p < 0.001; NE: not estimated

Multivariate model adjusted for age, sex, smoking, alcohol drinking, exercising, body mass index, duration of diabetes, age at diabetes diagnosis, types of diabetes treatment, complications, medication use, and biomarkers

## Discussion

In this study, a J-shaped association was found with increased all-cause and expanded CVD mortalities for sleep durations in people with T2D. The patients with sleep duration of between 5 and 7 h/day had the lowest all-cause and expanded CVD mortalities. The risk of all-cause and expanded CVD mortalities was higher in patients who sleep for less or more than 7 h per night and with age ≥ 65 years, diabetes duration ≥ 5 years, diabetes diagnosis ≤ 45 years, insulin use, SBP/DBP > 130/85 mmHg, TG ≥ 150 mg/dL, and HbA1c ≥ 7% than in those with 7 h of sleep and their counterparts. This study suggested that sleep duration exerts synergistic interaction effects across subgroups of diabetes duration for non-expanded CVD mortality and antagonistic interactions across age subgroups for expanded CVD mortality, and across obesity subgroups for all-cause and non-expanded CVD mortalities.

The associations between deviations from optimal sleep duration and a wide range of health risks may reflect the consequence of autonomic dysfunction due to sleep fragmentation, deterioration in quality, and micro-awakenings [37]. The autonomic nervous system plays an important role in the coordination of many important physiologic functions during sleep [38, 39]. Sleep disturbances may induce autonomic dysfunction [40] and exacerbate the metabolic control and thus lead to obesity [41], hypertension [42], impaired glucose tolerance [43], and insulin resistance [42, 43]. The biological consequences of sleep disturbances, including endocrine disorders [44], impaired immune function [44], oxidative stress [45], increasing inflammatory reactions, and endothelial dysfunction [45, 46], along with impaired physiology/diseases, may be involved in causing further adverse clinical outcomes, such as atherosclerosis and mortality [47]. These potentially biological mechanisms and impaired physiology and diseases may explain the findings in the present study, that is, short and long sleep durations are significant predictors of all-cause, expanded CVD, and non-expanded CVD mortalities. The joint effects of abnormal sleep pattern (sleep duration greater or shorter than 7 h) with age ≥ 65 years, duration ≥ 5 years, insulin use, SBP/DBP > 130/85 mmHg, TG ≥ 150 mg/dL, and poor glycemic control (HbA1c ≥ 7%) were associated with an increased risk of all-cause (HR 1.24–4.00) and expanded CVD (HR 1.25–3.82) mortalities. In addition, significant synergistic interactions were found between sleep duration and DM duration for non-expanded CVD mortality. By contrast, significant antagonistic interactions were observed between sleep duration and age for expanded CVD mortality, and between sleep duration and obesity (BMI ≥ 27 kg/m^2^) for all-cause and non-expanded CVD mortalities [48, 49]. The possible explanation for the antagonistic interaction between sleep duration and obesity is that obese patients with diabetes may receive better care and management, possess greater metabolic reserve against poor prognosis or outcomes [49], or acquire better prognosis or lower risk for complications and comorbidity than those with low BMI. A difference in disease etiology has been reported, that is, T2D in obesity is due to metabolic stress, which is associated with lower risk for complications and comorbidity [50], whereas diabetes in low BMI is due to genetic susceptibility [51].

Patients with T2D had the lowest risk of death at 7 h/day of sleep duration, similar to those observed in the general population [9, 31] and diabetic population [30, 33]. In addition, insufficient or excessive sleep was associated with all-cause and CVD mortalities in patients with T2D in the present study, showing a J-shaped relationship. When sex-specific associations were examined, short sleep duration associated with high risks of all-cause, expanded CVD, and non-expanded CVD mortalities was observed only in men but not in women, consistent with those reported in a USA study using data from the National Health Interview Survey [33]. However, this finding was contrary to that reported by a JACC Study [30]. The present study further reported the joint effects, demonstrating that the magnitude of associations between sleep duration and death was greater in patients with older age, longer diabetes duration, diabetes diagnosis at a younger age, hypertension, high TG, poor glycemic control, and insulin use than in their counterparts. Some of these joint effects supported the findings of a USA study, which revealed that the effects are pronounced for individuals diagnosed with early-onset diabetes, reported living with diabetes for a longer duration, and used oral glucose-lowering medication and insulin [33]. The present study further considered the joint effects of sleep duration and metabolic factors, such as TG and BMI, which were not accounted for by prior studies [26, 29]. The findings of the present study also suggested that monitoring of sleep duration in patients with T2D is warranted, particularly in those taking insulin, with early-onset diabetes, longer diabetes duration, poor glycemic control, and older age. Previous studies have not evaluated the biological interactions of sleep duration with metabolic factors, such as TG and BMI. The present study further assessed their modification effects on the survival of patients with diabetes. The results indicated that patients who had BMI < 27 kg/m^2^ and a longer or shorter sleep duration had 32%, 22%, and 40% higher risks of all-cause, expanded CVD, and non-expanded CVD mortalities, respectively, compared with those who had a normal sleep duration (7 h) and BMI < 27 kg/m^2^. However, patients with BMI ≥ 27 kg/m^2^ and a longer or shorter sleep duration did not encounter an increased risk of mortality. Future research is needed to elucidate the antagonistic interactions between sleep duration and obesity and the complex interplay between sleep duration and metabolic dysfunction (such as glycemia, hypertension, obesity, and dyslipidemia) to discern the pathophysiologic mechanisms linking their associations to mortality.

Disease status directly affecting sleep duration or abnormal sleep duration may contribute to poor disease conditions [52]. To rule out this possibility, our sensitivity analysis was performed in patients without those comorbidities. Even after excluding or adjusting for individuals with stroke, coronary artery disease, severe hypoglycemia, diabetic ketoacidosis, and hyperglycemic hyperosmolar nonketotic coma, a significant increase in all-cause and expanded CVD mortalities was still observed for patients with shorter or longer sleep duration (less than or equal to 4 h and greater than 9 h) and in non-expanded CVD mortality with longer than 9 h of sleep. Sensitivity analysis yielded similar results, indicating that the study findings are robust.

The findings of this study, including the independent effect of sleep duration and interaction effect with age; diabetes duration; and obesity on all-cause, expanded, and non-expanded CVD mortality, could provide information for health professionals to design sleep education intervention and identify persons at high risk, who could be targeted for sleep education intervention to reduce immature death. For future studies, the Mendelian randomization could be used to analyze the association of sleep duration with mortality to provide experimental evidence for causal inference.

## Strengths and limitations

This study has several advantages and limitations. Strengths included large sample size; a 10-year longitudinal follow-up; exclusion of participants who died within 3 years from baseline; data collection using standardized procedures; and precise definition of death from the National Death Datasets, which increased the validity of the results. Furthermore, three measures (RERI, AP, and S index) were used to estimate the direction of interactions of sleep duration and diabetes-related and metabolic factors in patients with T2D. Two limitations are worth mentioning. One is about sleep measurement. Sleep was measured by a self-reported question about typical sleep duration, not an objective measure. It lacks sufficient information on sleep quality and depression. And sleep duration during weekends or holiday is not considered. The other limitation is that although a significant increase in all-cause and expanded CVD mortalities was observed in patients with shorter than or equal to 4 h of sleep. These results should be interpreted with caution, because only 1.6% (n = 205) of the study subjects had sleep duration of less 4 h.

In conclusion, sleep duration is a significant predictor of all-cause, expanded CVD, and non-expanded CVD mortalities in patients with T2D. Sleep duration of 5–7 h had the lowest mortality risk, and a J-shaped association was observed. Significant synergistic interactions were found between sleep duration and age as well as diabetes duration, and antagonistic interactions were observed between sleep duration and obesity. These findings could provide information for health professionals to design sleep education intervention as well as to identify persons at high risk who could be targeted for sleep education intervention for reducing immature death.

## Supplementary Information

**Additional file 1: Figure S1.** Recruitment procedure.

**Additional file 2: Table S1.** Hazard ratios of mortality for sleep duration in patients with type 2 diabetes for sensitivity analysis by including those aged younger than 30 years and having sleep and related information (n = 12,833).

## Data Availability

The datasets generated and/or analyzed during the current study are not publicly available due to the policy declared by National Health Insurance in Taiwan but are available from the corresponding author on reasonable request.

## Abbreviations

NSF: National Sleep Foundation
RRs: Risk ratios
CVD: Cardiovascular disease
T2D: Type 2 diabetes
RERI: Relative excess risk due to interaction
AP: Proportion attributable to interaction
S index: Synergy index
DCMP: Diabetes Care Management Program
CMUH: China Medical University Hospital
ICD-9-CM: International Classification of Disease, 9th Revision, Clinical Modification
FPG: Fasting plasma glucose
TG: Triglyceride
HDL-C: High density lipoprotein-cholesterol
LDL-C: Low density lipoprotein-cholesterol
TC: Total cholesterol
OSA: Obstructive sleep apnea
BZD: Benzodiazepine
BMI: Body mass index
SBP: Systolic blood pressure
DBP: Diastolic blood pressure
CVs: Coefficient variations
eGFR: Estimated glomerular filtration rate
HHNK: Hyperglycemia hyperosmolar non-ketoacidosis
DKA: Diabetic ketoacidosis
ICD-10-CM: International Classification of Disease, Tenth Revision, Clinical Modification
K–M: Kaplan–Meier
HRs: Hazard ratios
CIs: Confidence intervals
