# Supplementary material for: Sleep duration predicts subsequent long-term mortality in patients with type 2 diabetes: a large single-center cohort study
Source: Cardiovasc Diabetol. 2022 Apr 27;21:60. doi: 10.1186/s12933-022-01500-0 (PMC9045470; doi:10.1186/s12933-022-01500-0)
Supplement: Supplementary file 1 — Additional file 1: Figure S1. Recruitment procedure. [file 12933_2022_1500_MOESM1_ESM.docx]

A total of 18,373 adults were enrolled in the Diabetes Care Management Program.

A total of 12,526 participants were included for analysis.

A total of 16,414 subjects were eligible.

Exclusion criteria:

Type 1 diabetes or gestational diabetes (*n* = 448)

Age < 30 years (*n* = 504)

Enrollment period for NDCMP less than 3 years (*n* =1,007)

Without sleep information (*n* = 3,137)

Without sociodemographic factors, lifestyle behaviors, diabetes-related factor, complications, and blood biochemical indices (*n* = 751)

**Figure S1.** Recruitment procedure
