# Supplementary material for: Sleep duration predicts subsequent long-term mortality in patients with type 2 diabetes: a large single-center cohort study
Source: Cardiovasc Diabetol. 2022 Apr 27;21:60. doi: 10.1186/s12933-022-01500-0 (PMC9045470; doi:10.1186/s12933-022-01500-0)
Supplement: Supplementary file 2 — Additional file 2: Table S1. Hazard ratios of mortality for sleep duration in patients with type 2 diabetes for sensitivity analysis by including those aged younger than 30 years and having sleep and related information (n = 12,833). [file 12933_2022_1500_MOESM2_ESM.docx]

**Table S1.** Hazard ratios of mortality for sleep duration in patients with type 2 diabetes for sensitivity analysis by including those aged younger than 30 years and having sleep and related information (n = 12,833)

| Variables | HR (95% CI) | | |
| --- | --- | --- | --- |
|  | All-cause mortality | Expanded CVD mortality | Non-expanded CVD mortality |
| ***Sleep duration (h/day)*** |  |  |  |
| ≤ 4 | 1.41 (1.06, 1.86)* | 1.53 (1.04, 2.27)* | 1.27 (0.85, 1.89) |
| 5–6 | 1.06 (0.94, 1.20) | 1.02 (0.85, 1.23) | 1.09 (0.92, 1.29) |
| 7 | 1.00 | 1.00 | 1.00 |
| 8 | 1.10 (0.99, 1.23) | 1.04 (0.89, 1.23) | 1.15 (1.00, 1.33) |
| 9–10 | 1.37 (1.24, 1.52)*** | 1.34 (1.15, 1.57)*** | 1.40 (1.21, 1.61)*** |
| > 10 | 1.83 (1.56, 2.15)*** | 1.90 (1.50, 2.39)*** | 1.76 (1.40, 2.22)*** |

Multivariate model adjusted for age, sex, smoking, alcohol drinking, exercising, body mass index, duration of diabetes, age at diabetes diagnosis, types of diabetes, complications, medication use, and biomarkers.

*: *p* < 0.05; ***: *p* < 0.001.
